# Emotional intelligence predicts individual differences in proneness for flow among musicians: the role of control and distributed attention

**DOI:** 10.3389/fpsyg.2014.00608

**Published:** 2014-06-17

**Authors:** Narayanan Srinivasan, Bruno Gingras

**Affiliations:** ^1^Centre of Behavioural and Cognitive Sciences, University of AllahabadAllahabad, India; ^2^Department of Cognitive Biology, Faculty of Life Sciences, University of ViennaVienna, Austria

**Keywords:** flow, music, emotional intelligence, control, hierarchical control, distributed attention, positive emotions

The experience of flow, or optimal experience (Csikszentmihalyi, 1990, 2002), is an intensely rewarding psychological state that has been linked with peak performance and high achievement in a variety of settings ranging from competitive sports to the workplace. While the concept of flow remains somewhat elusive, its study has broad implications for training as well as for pedagogical and motivational strategies. Although flow has been studied for more than 30 years, little is known about why some people are more prone to attain flow states than others (also known as “autotelic personalities”), and Marin and Bhattacharya (2013) are the first to address this issue in the field of music performance. In their study, they investigated the relationship between several factors, ranging from personality traits to amount of daily practice and age of first piano lessons, and the frequency and intensity with which pianists experienced flow states. Here, we review their main findings and discuss the potential role of control and distributed attention in the experience of flow in music performance.

As pointed out by the authors, flow in music performance should not necessarily be equated with flow in other activities given the importance of emotions in musical communication (Juslin and Sloboda, 2010). Thus, although emotion is not a chief component of Csikszentmihalyi's multidimensional concept of flow (besides the intense happiness associated with flow states), Marin and Bhattacharya surmised that the affective tone of a musical piece, as well as performers' emotional intelligence, might play an important role in flow in music performance.

Piano students at the postgraduate and undergraduate levels were invited to fill a questionnaire, including standardized tests on flow (Jackson and Eklund, 2004) and trait emotional intelligence (Petrides and Furnham, 2006), as well as a series of questions covering musical preferences, musical emotions, amount of musical training, and socio-demographic variables. Daily amount of practice and trait emotional intelligence were found to be significant predictors of flow experiences, whereas age of first piano lessons, years of music training, age, and gender were not predictive. The finding that flow was positively correlated with emotional intelligence is in line with Goleman's (1995) theory. However, flow was not correlated with high achievement in piano performance, as measured by the likelihood of having won a prize in piano competitions. Nevertheless, we should point out that musical achievement is not easy to define objectively and other measures of achievement may need to be taken into account.

Interestingly, the music of some composers, notably Romantic composer Frédéric Chopin, was reported by pianists to induce flow states more reliably than others. More generally, pianists tended to experience flow when playing music in a familiar musical style, and especially from their favorite style, suggesting a relationship between familiarity, preference, and flow.

The cognitive and affective processes that lead to flow are important aspects that need further exploration, given that they may lend some insight into the putative link between emotional intelligence and flow. More specifically, two aspects need to be probed further in terms of flow in musicians. The first such aspect is control. Successful control depends on prediction of outcomes of actions in controlling perception. In terms of personality, this may depend on higher emotional intelligence that might involve an internal locus of control, which emphasizes preparation and effort. The event-control approach argues for the presence of hierarchically organized control loops at multiple spatiotemporal scales and people's feeling about self and agency depending on the highest level at which control is achieved (Jordan, 2003; Kumar and Srinivasan, 2014). Control exercised at a higher level depends on control exercised at lower levels in the hierarchy. An inexperienced musician attends mostly to the perceptual-motor control (fingers and keys) needed to play a musical piece accurately whereas an expert musician focuses mainly on the overarching musical structure and the emotions conveyed through the piece. In the context of the event-control approach, the musical structure or emotions would be linked to control at the higher level and the actual movements made by the performer would be linked to control at the lower level. Thus, attaining a flow state during a musical performance would depend on achieving control at all levels in the control hierarchy, which might be easier to achieve for performers with higher emotional intelligence or an internal locus of control (Keller and Blomann, 2008). The successful control achieved in playing a piece would result in happiness especially among performers with a perceived internal locus of control, given the links between internal locus of control and happiness (Lu, 1999; Pannells and Claxton, 2008). Consistent with this model, Marin and Bhattacharya reported that among the nine dimensions of the flow construct measured by Jackson and Eklund's (2004) questionnaire, “sense of control” was the second most highly correlated with the average global flow score in pianists, after “autotelic experience.”

A second important aspect is the role of attention and its potential link to emotions (Fredrickson, 2004; Srinivasan and Hanif, 2010; Srinivasan and Gupta, 2011). Positive emotions have been linked to distributed or global attention, whereas negative emotions have been linked to focused or local attention. One possible way in which attention might play a role in terms of event-control is the manner in which attention is efficiently distributed across multiple levels in a control hierarchy. Too much focused attention on any one level could result in fewer positive emotions leading to a reduced flow experience. This may explain why winning a performance prize is not necessarily linked to flow experience. The focus needed to avoid distractions and perform well in competitions may help in achieving a good performance given the extra focused attention, but at the cost of increased negative emotions, possibly preventing performers from attaining a flow state.

Combining these two aspects, we posit that, for performers to reach a flow state, attention needs to be effortlessly distributed across multiple levels in the control hierarchy, while successfully retaining control at all levels. This would thus lead to positive emotions, further reinforcing the motivation to practice and play more music.

## Conflict of interest statement

The authors declare that the research was conducted in the absence of any commercial or financial relationships that could be construed as a potential conflict of interest.
